# Prognostic and therapeutic implications of clinical-radiologic discrepancy in parametrial invasion prior to primary radical hysterectomy in cervical cancer

**DOI:** 10.1016/j.gore.2025.102002

**Published:** 2025-12-09

**Authors:** Ester P. Olthof, Nicholai A. Oostveen, Maaike A. van der Aa, Ruud L.M. Bekkers, Constantijne H. Mom, Jacobus van der Velden, Joost Nederend, Edith M.G. van Esch

**Affiliations:** a Department of Research and Development, Netherlands Comprehensive Cancer Organization, Utrecht, the Netherlands; b Department of Gynecologic Oncology, Cancer Center Amsterdam, Amsterdam University Medical Centre, Centre for Gynecologic Oncology Amsterdam (CGOA), Amsterdam, the Netherlands; c Department of Obstetrics and Gynecology, Catharina Hospital, Eindhoven, the Netherlands; d Department of Obstetrics and Gynecology, Radboudumc, Nijmegen, the Netherlands; e GROW School of Medicine, Maastricht, the Netherlands; f Department of Radiology, Catharina Hospital, Eindhoven, the Netherlands; g Department of Gynecologic Oncology, Erasmus Medical Center, Erasmus MC Cancer Centre, Rotterdam, the Netherlands

**Keywords:** Cervical cancer, Neoplasm Staging, FIGO classification, TNM staging, Magnetic Resonance Imaging

## Abstract

**Aim:**

This study evaluates the prognostic and therapeutic implications of clinical-radiologic discrepancy in parametrial invasion of cervical cancer prior to radical hysterectomy. We compared patients with radiological presence but clinical absence of parametrial invasion (discrepancy group) to those without radiologic and clinical suspicion of parametrial invasion (consensus group).

**Methods:**

Women with International Federation of Gynaecology and Obstetrics (2009) stage IA-IIA cervical cancer, diagnosed between 2009 and 2017, who underwent magnetic resonance imaging prior to radical hysterectomy were retrospectively selected from the Netherlands Cancer Registry. Kaplan-Meier estimates and Cox proportional hazards were used for survival and logistic regression for risk of adjuvant therapy and toxicity.

**Results:**

Of 886 patients included, 87 (10%) had clinical-radiologic parametrial invasion discrepancy. Patients with discrepancy were more likely to have poor prognostic factors (i.e., a larger tumor, increased depth of invasion, lymphovascular space invasion, nodal metastases and positive resection margins) than those without. The 5-year disease-free and overall survival rates were lower in the discrepancy (74% and 82%) than in the consensus group (86% and 92%). However, after confounder adjustments, disease-free and overall survival were not affected by clinical-radiologic discrepancy. Patients with discrepancy in parametrial invasion were more likely to receive adjuvant therapy (54% vs 23%) and experience therapy-related toxicity (44% vs 29%).

**Conclusion:**

Clinical-radiologic discrepancy of parametrial invasion occurs in approximately 10% of patients and is associated with poor prognostic factors and increased likelihood of adjuvant therapy and toxicity. This highlights the importance of addressing these factors in treatment counselling for either primary chemoradiotherapy or surgery.

## Highlights

• Clinical-radiologic discrepancy of parametrial invasion in cervical cancer is a therapeutic dilemma.
• Discrepancy in parametrial invasion at diagnosis is associated with poor prognostic factors of cervical cancer.
• Discrepancy of parametrial invasion is associated with positive resection margins and increased multimodality treatment.
• Patients with discrepancy of parametrial invasion at diagnosis experience more therapy-related toxicity (44% vs 29%)
• Future research on optimalisation of MRI diagnostics and value of clinical examination (under anesthesia) is important.

## 1 Introduction

Cervical cancer is the fourth most common cancer in women worldwide, with an estimated 604,000 new cases reported in 2020 (Sung et al., 2021). Cervical cancer can spread by local extension to the parametria, known as parametrial invasion (PMI), corresponding to International Federation of Gynecology and Obstetrics (FIGO) stage IIB (Bhatla et al., 2018) PMI is an important parameter for staging patients and determining appropriate treatment. Presence of PMI may direct primary treatment from (radical) surgery to pelvic radiotherapy with concurrent (cisplatin-based) chemotherapy (Bhatla et al., 2018, Cibula et al., 2023). Additionally, presence of PMI in postoperative resection specimens after radical hysterectomy is considered a pathologically proven high-risk factor and therefore, an indication for adjuvant chemoradiotherapy (Cibula et al., 2023). As a result, these patients receive both surgery and chemoradiotherapy, known as multimodality therapy. The ESGO/ESTRO/ESP guidelines recommend avoiding multimodality therapy because of the increased morbidity associated with combined treatment (Bhatla et al., 2018). If risk factors requiring adjuvant treatment are known at the time of diagnosis, definitive chemoradiation therapy may be considered without prior radical pelvic surgery (Bhatla et al., 2018).

PMI can be diagnosed by clinical examination and/or magnetic resonance imaging (MRI). Current guidelines recommend a pelvic MRI to assess local tumor extension (Cibula et al., 2023). MRI has reported sensitivity and specificity rates of 76–84 % and 92–94 %, respectively, exceeding the 40 % sensitivity and matching the specificity of clinical examination (Thomeer et al., 2013, Woo et al., 2018). However, evaluation can be difficult when abnormalities are more subtle. Furthermore, the lack of a reliable gold standard (pathological confirmation) in many studies complicates the interpretation of the results. There's a notable false positive rate, with one prospective study showing a positive predictive value of MRI ranging from 23 % to 50 % (Mongula et al., 2019). Recent data suggest that MRI staging could alter treatment plans in 12 % of cases, but there is a risk of overtreatment due to potential false-positive results (Smits et al., 2023).

Currently, there is limited evidence regarding the optimal treatment regimen for patients without parametrial invasion at clinical examination but with radiologic upstaging due to PMI on MRI. Therefore, this retrospective cohort study evaluates the prognostic and therapeutic implications of clinical-radiologic discrepancy in PMI of cervical cancer prior to radical hysterectomy, by comparing patients with radiologic suspicion of PMI to those without. Importantly, this study does not assess the diagnostic accuracy of clinical examination or MRI staging in detecting PMI, as our study population and design may induce bias. Instead, we aim to determine whether clinical examination or radiologic staging by MRI should be prioritized in guiding treatment decisions for patients with clinically absent but radiologically suspected PMI.

## 2 Material and methods

### 2.1 Study design and data collection

We conducted a nationwide retrospective cohort study by analyzing data from the Netherlands Cancer Registry (NCR) after approval of the Privacy Review Board (No. 23094) of the Netherlands Cancer Registry. The NCR is a population-based registry covering all newly diagnosed malignancies in the Netherlands. We included women with (1) squamous, adenosquamous, or adenocarcinoma of the cervix (2) diagnosed between January 2009 and January 2017, (3) an age of ≥ 18 years, (4) treated with radical hysterectomy and lymphadenectomy (regardless of adjuvant therapy), and (5) who had pretreatment imaging by MRI. Patients with: (1) clinical suspicion of PMI, (2) an unknown pathological status of PMI, or (3) adjuvant chemotherapy only were excluded. Generally, adjuvant (chemo)radiotherapy was indicated in the case of postoperative intermediate or high-risk factors and administered according to local protocols: pelvic external beam radiotherapy (i.e., 45–50 Gy) and concurrent chemotherapy (i.e., cisplatin 40 mg/m2 weekly) (Thomeer et al., 2013, Yoon et al., 2016). Patients were categorized and compared based on discrepancy or consensus between MRI and clinical examination results concerning PMI: patients with clinical absence of PMI, but presence of PMI on MRI (discrepancy group) were compared with patients who had consensus on absence of PMI (consensus group).

Clinicopathological and treatment characteristics, including toxicity, were collected by trained data managers from the NCR. Pathological PMI was defined according to local protocols (i.e., presence of direct invasion, a lymph node metastasis, and/or lymphovascular space invasion in the parametria). The pathological PMI status was recorded from patient records as reported by the pathologist, categorized as (0) not reported; (1) reported, no invasion; (2) reported; invasion. The PMI status on MRI was recorded from patient records as reported by the radiologist and categorized as (0) definitely not; (1) definitely; (2) probably not; (3) probably (suspicious); (9) unknown. This classification was also used to define the clinical status of PMI, determined by examination under anesthesia (EUA) or outpatient clinical examination. A “definitely” and “probably (suspicious)” status were considered positive, while “definitely not” and “probably not” were considered negative for PMI. During the study period, no standardized national guidelines existed for the reporting of MRI findings in cervical cancer cases, nor for the acquisition of the images. Surgery-related toxicity was defined as grade ≥ 2 of the Clavien-Dindo scale within 30 days after surgery (Clavien et al., 2009). Toxicities associated with radiotherapy and chemotherapy were defined as grade ≥ 3 according to the Common Terminology Criteria for Adverse Events (CTCAE; version 4.0.2) within 6 months after initiating treatment (Health, N.I.o., Common terminology criteria for adverse events (CTCAE) v4. 0. (No Title), 2010).

### 2.2 Outcomes and definitions

Primary endpoints were disease-free survival (DFS), defined as time between radical hysterectomy and recurrence, and overall survival (OS), defined as time between diagnosis and death. The date of death was obtained by annually linking to the Municipal Personal Records Database, latest update 1st of January 2023. Patients’ recurrence status was obtained from hospital records. Patients without a recurrence, or lost to follow-up, were censored at the last date of clinical contact. Secondary endpoints were the risk of adjuvant therapy (i.e., radiotherapy, chemotherapy, or both) and the risk of therapy-related toxicity. Pathologic PMI was defined by local protocols as direct tumor extension into the parametria and/or the presence of lymph node metastases in the parametria.

### 2.3 Statistical analysis

For descriptive statistics, continuous variables were tested on normality by Shapiro-Wilk tests and analyzed using the Mann-Whitney U or unpaired *t*-test, while categorical variables were analyzed using the Fisher’s exact test. Unadjusted survival analyses were performed using the Kaplan-Meier method with log-rank testing. The multivariable Cox proportional hazards method was used for adjusted survival analysis. The proportional hazards assumption was tested by plotting Schoenfeld residuals, no violations were found. Uni- and multivariable logistic regression analyses were used to assess the risk of adjuvant therapy according to parametrial status at MRI and the risk of therapy-related toxicity. All analyses were performed using Stata/SE, version 17.0 (Stata Corporation, College Station, TX, USA), a p-value < 0.05 was considered significant.

## 3 Results

### 3.1 Patient characteristics

**Fig. 1:**
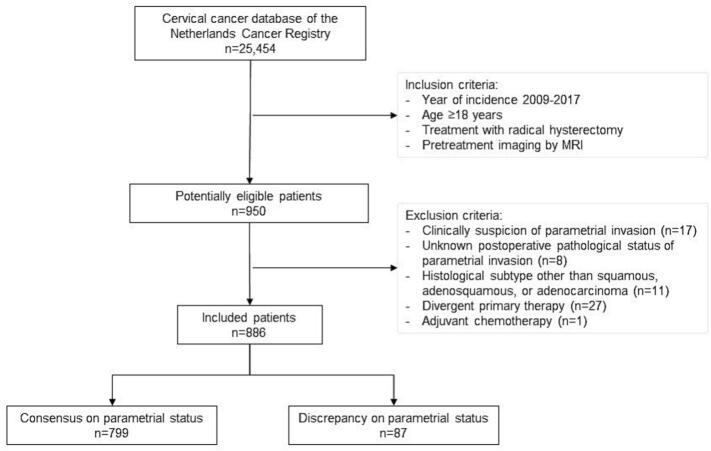
Patient Selection Flow Chart.

A total of 886 patients were included (Fig. 1), all without clinical suspicion of PMI. Of these patients, 10 % had suspected PMI on MRI (discrepancy group; n = 87). Baseline characteristics of the consensus (n = 799) and discrepancy groups are presented in Table 1. Patients in the discrepancy group were more likely to be older (46 versus 44 years; p = 0.028). The discrepancy group also had worse prognostic factors compared to the consensus group, such as FIGO 2009 stage IB2-IIA2 (33 % versus 11 %; p < 0.001), larger clinical tumor size (36 versus 20 mm; p < 0.001), presence of pathological lymph node metastasis (26 % versus 15 %; p = 0.01), invasion depth > 5 mm (86 % versus 62 %; p < 0.001) and presence of lymphovascular space invasion (60 % versus 44 %; p = 0.008). Pathological parametrial involvement was observed in 9 % in the discrepant and 4 % in the consensus group (p = 0.057).

**Table 1:** Patient characteristics.

**Characteristics**	**Missing**		**Discrepancy (n = 87)**	**Consensus (n = 799)**	**p-value**
Age†, years			46 [26–79]	44 [22–82]	0.028*
BMI†, kg/m2	24		24 [19–36]	25 [16–49]	0.92
Charlson comorbidity index	142				0.68
0			63 (84 %)	567 (85 %)	
1			9 (12 %)	84 (13 %)	
≥2			3 (4 %)	18 (3 %)	
FIGO 2009 stage					<0.001*
IA			0 (0 %)	13 (2 %)	
IB1			58 (67 %)	705 (88 %)	
IB2			14 (16 %)	44 (6 %)	
IIA1			8 (9 %)	32 (4 %)	
IIA2			7 (8 %)	5 (1 %)	
Tumor size†					
Clinical, mm‡	48		36 [0–73]	20 [0–70]	<0.001*
Pathological, mm	138		35 [9–73]	21 [1–80]	<0.001*
Lymph node status,					
pN1			23 (26 %)	123 (15 %)	0.01*
Histological subtype	2				0.050
Squamous cell carcinoma			68 (78 %)	524 (66 %)	
Adenocarcinoma			15 (17 %)	232 (29 %)	
Adenosquamous carcinoma			4 (5 %)	43 (5 %)	
Depth of invasion	70				<0.001*
<3mm			6 (8 %)	114 (15 %)	
3–5 mm			5 (6 %)	164 (22 %)	
>5mm			66 (86 %)	461 (62 %)	
LVSI, yes	49		50 (60 %)	333 (44 %)	0.008*
Differentiation grade	248				0.04*
1			5 (9 %)	71 (12 %)	
2			23 (40 %)	311 (54 %)	
3			30 (52 %)	198 (34 %)	
Pathological parametrial involvement, yes			8 (9 %)	34 (4 %)	0.057
Positive surgical margins, yes	4		6 (7 %)	27 (3 %)	0.13
Surgical approach					0.001*
Open			74 (85 %)	524 (66 %)	
Laparoscopic			2 (2 %)	64 (8 %)	
Robot-assisted			11 (13 %)	211 (26 %)	
Adjuvant therapy, yes			47 (54 %)	180 (23 %)	<0.001*
Chemoradiotherapy			14 (16 %)	94 (12 %)	
Radiotherapy			33 (38 %)	86 (11 %)	
Recurrence, yes			22 (25 %)	110(14 %)	0.007*
All-cause mortality, yes			20 (23 %)	96 (12 %)	0.007*

Data are the number of patients (percentage) or median [range].

Due to rounding some totals do not add up to 100%.

*Statistically significant.

†Continuous scale.

‡Clinical tumor size is MRI based and supplemented with size based on physical examination when missing.

*Abbreviations:* BMI, body mass index; FIGO, International Federation of Gynecology and Obstetrics; pN1, pathological positive node status; LVSI, lymphovascular space invasion.

### 3.2 Survival outcomes

**Fig. 2:**
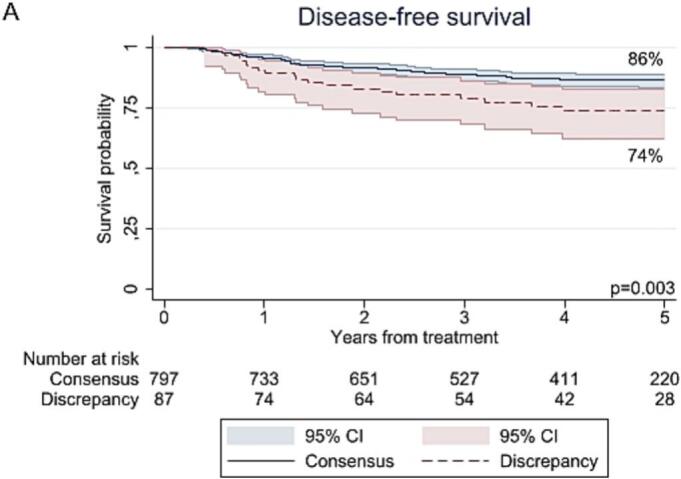

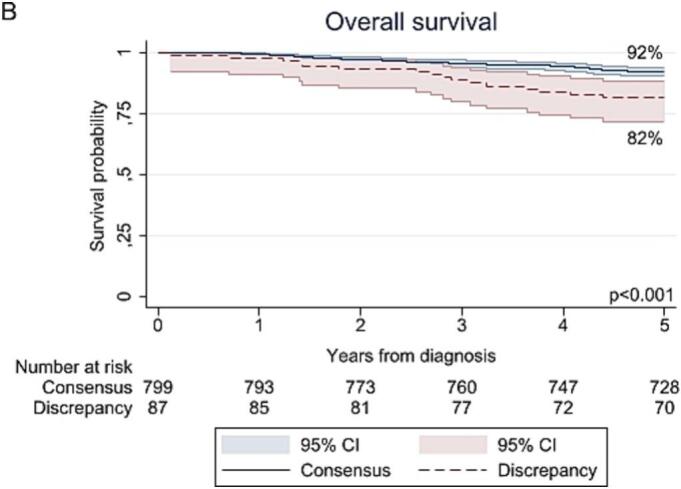
Kaplan-Meier curves for (A) recurrence-free survival and (B) overall survival.

The median follow-up time for DFS and OS was 49 months (95 % CI 0–138) and 95 months (95 % CI 1–169), respectively, and did not differ between the consensus and discrepancy group. The rates of recurrence (25 % versus 14 %; p = 0.007) and mortality (23 % versus 12 %; p = 0.007) were higher in the discrepancy group than the consensus group. As shown in Fig. 2, the 5-year DFS and OS were worse in the discrepancy group compared to the consensus group, with rates of 74 % versus 86 % (p = 0.003) and 82 % versus 92 % (p < 0.001). However, after adjustment for confounders (i.e., age, FIGO stage, primary tumor size, pathological lymph node status, histological subtype, LVSI, pathological parametrial involvement, positive surgical margins), suspicion of PMI on MRI was no longer associated with a worse DFS (HR 1.00; 95 % CI 0.60–1.66; p = 0.99) nor OS (HR 0.94; 95 % CI 0.55–1.62; p = 0.82) (Supplementary Table S1).

### 3.3 Risk of adjuvant therapy

The rate of administration of adjuvant therapy in the discrepancy group (54 %; n = 47) was twice that of the consensus group (23 %; n = 180) (p < 0.001), consisting mainly of radiotherapy (38 %; n = 33). Patients with clinical-radiologic discrepancy were more likely to receive adjuvant therapy (odds ratio (OR) 3.10; 95 % CI 1.56–6.16; p = 0.001), while adjusting for potential confounders (i.e., age, primary tumor size, pathological lymph node status, depth of invasion, LVSI, positive surgical margins, pathological parametrial involvement) (Supplementary Table S2).

### 3.4 Therapy related toxicity

As shown in Table 2, the discrepancy group (44 %; n = 38) experienced more treatment-related toxicity than the consensus group (29 %; n = 232) (p = 0.007). This was caused by accumulation of higher surgery (39 % versus 27 %; p = 0.024) and radiotherapy-related toxicity rates (5 % versus 1 %; p = 0.031) in the discrepancy than consensus group, whereas the chemotherapy-related toxicity rates were comparably low (1 % and 2 %; p = 1.00).

**Table 2:** Therapy-related toxicities.

		**Discrepancy**	**Consensus**	**p-value**
**Surgery**				
Intraoperative injury		4 (5 %)	35 (4 %)	0.79
Infection		10 (11 %)	52 (7 %)	0.12
Trombo-embolic event		2 (2 %)	2 (0 %)	0.050*
IC-admission		1 (1 %)	7 (1 %)	0.56
Bladder function disorder		20 (23 %)	105 (13 %)	0.022*
Blood transfusion		7 (8 %)	37 (5 %)	0.19
Other		4 (5 %)	21 (3 %)	0.30
Total events†		48	256	
Total patients		34 (39 %)	218 (27 %)	0.024*
				
**Radiotherapy**				
Urological		1 (1 %)	2 (0 %)	0.27
Gastrointestinal		4 (5 %)	7 (1 %)	0.017*
Other		1 (1 %)	1 (0 %)	0.19
Total events†		6	10	
Total patients		4 (5 %)	9 (1 %)	0.031*
				
**Chemotherapy**				
Nausea/anorexia		0 (0 %)	3(0 %)	1.00
Vomiting		0 (0 %)	3 (0 %)	1.00
Nephrotoxicity		0 (0 %)	2 (0 %)	1.00
Ototoxicity		0 (0 %)	0 (0 %)	1.00
Stomatitis		0 (0 %)	1 (0 %)	1.00
Bone marrow depression		0 (0 %)	1 (0 %)	1.00
Malaise/fatigue		0 (0 %)	2 (0 %)	1.00
Neurotoxicity		0 (0 %)	2 (0 %)	1.00
Other		4 (1 %)	3 (0 %)	0.34
Total events†		1	19	
Total patients		1 (1 %)	13 (2 %)	1.00
				
**Total adverse events**				
Total events†		55	285	
Total patients		38 (44 %)	232 (29 %)	0.007*

* Statistically significant.

† Some patients experienced multiple toxicities.

## 4 Discussion

Clinical-radiologic discrepancy of PMI in cervical cancer is a clinical controversy in the diagnostic work-up. This study highlights the importance of radiologic suspicion of PMI, which occurs in approximately 10 % of patients with clinically absence of PMI. Radiologic suspicion of PMI is associated with poor prognostic factors and a 3-fold increased risk of adjuvant therapy, especially radiotherapy, and more therapy-related toxicity caused by the accumulation of surgery and radiotherapy related toxicity rates.

We describe a 5-year DFS and OS of approximately 90 % in cases with clinical and radiologic absence of parametrial, which is consistent with other studies (Wenzel et al., 2020, Jensen et al., 2020, Bizzarri and Querleu, 2023). However, the 5-year survival rates for our discrepancy group are significantly lower (74 % and 82 %, respectively) in univariable analysis, though not after adjusting for confounders. These data suggest that the poorer oncological outcomes seen in cases with radiologic suspicion of PMI are associated with poor predictive factors despite adjuvant therapy (Cibula et al., 2023, Fuller et al., 1989). LVSI, depth of invasion, older age, tumor size, positive pelvic lymph nodes, and ovarian metastasis have previously been associated with pathologically proven PMI (Kodama et al., 2011, Xie et al., 2016). This raises the question whether patients with radiologic suspicion of PMI should be classified as high-risk for single surgical treatment failure with significant risk of adjuvant therapy.

Radiologic suspicion of PMI was an independent risk factor (OR 3.1) for adjuvant therapy after adjusting for pathologically proven combined high- and intermediate-risk factors. Moreover, half of our patients with clinical-radiologic discrepancy required adjuvant therapy, twice as many as in the consensus group (54 % vs. 23 %). The incidence of adjuvant therapy in the consensus group matches the rate of 24 % in a Dutch study after open or laparoscopic radical hysterectomy (Wenzel et al., 2020). Previous data in a discrepancy group treated by radical hysterectomy with lymph node dissection reported 83 % patients receiving adjuvant therapy (44 % chemoradiotherapy, 36 % radiotherapy and 1 % chemotherapy) (Yoon et al., 2016). Translated to clinical practice, in case of clinical-radiologic discrepancy, the presence of poor prognostic factors should be discussed in a multidisciplinary team and with the individual patient to counsel the advantages and disadvantages of either surgical therapy with increased risk of adjuvant (chemo)radiotherapy or primary chemoradiotherapy. If surgery is preferred, stepwise surgery with evaluation of pelvic lymph nodes prior to radical hysterectomy may be useful to avoid multimodality therapy, as clinical-radiologic discrepancy of PMI is associated with an increased risk of lymph node metastases (from 15 % to 26 % in this study). Sentinel lymph node resection, either minimally invasive with or without frozen section, can be performed (Cibula et al., 2023, Cibula et al., 2020, Berasaluce Gómez et al., 2023, Cibula et al., 2024). However, PMI is also correlated with the risk of non-sentinel lymph node metastasis (Diniz et al., 2021).

Therapy-related toxicities were increased in the clinical-radiologic discrepancy group (44 % vs 29 %) and were mainly surgical-related (91 %). We reasoned this, at least in part, to be explained by mainly laparotomy procedures and the radicality of surgery according to the Querleu-Morrow classification (Derks et al., 2016) due to larger tumorsize. In the retrospective study design we did not gather enough data to provide a robust statement on the impact of multimodality treatment for patients in cervical cancer therapy, we only registered the short-term toxicities by using a cut-off of six months and data lack standardized postoperative protocols. The incidences of chemo- and radiotherapy-related toxicity are relatively low in this study cohort. In literature, the health related quality of life in early stage cervical cancer is lower if surgery is followed by radiotherapy (Suvannasarn et al., 2022, Pfaendler et al., 2015, Greimel et al., 2009). Although results are conflicting, it appears that long-term morbidity, health related quality of life, and sexual functioning are affected differently by primary (chemo)radiotherapy (e.g., worse sexual functioning) compared to radical hysterectomy ± adjuvant therapy (e.g., more lymphoedema), advocating for shared decision-making and personalized treatment (Derks et al., 2017, Wenzel et al., 2022).

This study enables a more thorough understanding of the long-term outcomes and implications of clinical-radiologic discrepancies in cervical cancer treatment, utilizing comprehensive, real-life national data. However, there are notable limitations. At first, the study cohort focused on clinical absent PMI and radiological discrepancies on MRI of suspected PMI, to evaluate if clinical or radiological staging should be prioritized. The study excludes another relevant discrepant group thought; the group with clinical positive PMI and radiological positive or negative PMI, because this group lacks pathological confirmation of PMI which was a inclusion criteria for this study. This selection bias might withhold clinical outcomes from this specific, in general more locally advanced subgroup, treated with chemoradiation. Another limitation of this study is the lack of detailed information on the characteristics and quality of the MRI scans used, as it is known that variability in quality of both acquisition as reports exists and influence accuracy (Thomeer et al., 2013). This lack of data may affect the interpretation and generalizability of the findings. In our cohort with clinical absence of PMI, the incidence of pathologically confirmed PMI detected by MRI was only 9 %. This low positive rate is clearly influenced by our study population, which consisted exclusively of patients eligible for surgical intervention. As mentioned before, we were therefore unable to determine the diagnostic accuracy of clinical and/or MRI staging for PMI. However, we can compare the PMI positive rate with other surgically treated cohorts that report PMI proportions of 9–41 % (Sodeikat, P., Lia, M., Martin, M., The Importance of Clinical Examination under General Anesthesia: Improving Parametrial Assessment in Cervical Cancer Patients. Cancers (Basel), 2021, Canaz et al., 2017).

Furthermore, only information on presence or absence of PMI on MRI is available and no Likert scales on whether there is suspicion of PMI on MRI or not. This retrospective cohort provides no information regarding the bases on what the decision-making was done, nor we have information about the composition of the multidisciplinary team (e.g. was there a radiologist present). Also, the order of MRI and clinical examination is unknown, as known result of the first exam might influence the results of the second. It is imperative to reassess the positive predictive value of MRI for parametrial invasion using contemporary, state-of-the-art scanners and protocols (Manganaro et al., 2021). This reevaluation is crucial to determine the reliability of solely using radiologic FIGO staging by MRI in clinical practice. The significant shift in cervical cancer staging with the inclusion of imaging in the 2018 FIGO system highlights the importance of this assessment. Previous retrospective data indicate that diagnosis of patients without PMI is improved by MRI with a NPV of 76.9 % compared to 65.3 % NPV in EUA (Bleker et al., 2013). Therefore, in the case of inconclusive outpatient clinical staging, pretreatment staging may be limited to MRI without EUA. Another proposed method to improve accuracy of the per-operative assessment of PMI is combining the results of EUA in certain patient groups. Sodeikat et al. describe the additional value of EUA with the MRI, defined as augmented EUA (when the MRI was available during EUA), compared to MRI alone, especially in the tumors > 2.5 cm, improving accuracy from 76 % to 83 % (Sodeikat, P., Lia, M., Martin, M., The Importance of Clinical Examination under General Anesthesia: Improving Parametrial Assessment in Cervical Cancer Patients. Cancers (Basel), 2021). In this study cohort we are not able to conclude if clinical or radiological examination on PMI should be leading in clinical decision making. Though these data on discrepancy which indicates high-risk for single surgical treatment failure with significant risk of adjuvant therapy, highlight the importance of discussing these factors in multidisciplinary board and treatment counselling settings.

Future research should focus on optimizing MRI diagnostics, the value of augmented clinical examination/EUA and the impact of (stepped) surgery in case of clinical-radiologic discrepancy on PMI to determine whether primary chemoradiotherapy or (stepped) surgery should be the preferred treatment option for these patients evaluating both overall or recurrence free survival, multimodality therapy and impact in health-related quality of life.

## 5 Conclusions

In conclusion, clinical-radiologic discrepancy is a difficult clinical dilemma, particularly for patients with PMI detected on MRI but not by clinical examination. Such discrepancies, where radiologic suspicion is present without corresponding clinical evidence, occur in approximately 10 % of patients undergoing radical hysterectomy and seems to be associated with poor prognostic factors and increased likelihood of adjuvant therapy and toxicity. This highlights the importance of addressing these factors in treatment counselling of either primary chemoradiotherapy or surgery.

## CRediT authorship contribution statement

**Ester P. Olthof:** Writing – review & editing, Writing – original draft, Visualization, Methodology, Investigation, Formal analysis, Data curation, Conceptualization. **Nicholai A. Oostveen:** Writing – original draft, Visualization, Software, Investigation, Formal analysis. **Maaike A. van der Aa:** Writing – review & editing, Supervision, Funding acquisition, Conceptualization. **Ruud L.M. Bekkers:** . **Constantijne H. Mom:** Writing – review & editing, Methodology, Funding acquisition, Conceptualization. **Jacobus van der Velden:** Writing – review & editing, Methodology, Funding acquisition, Conceptualization. **Joost Nederend:** Writing – review & editing, Writing – original draft, Visualization, Supervision, Methodology, Conceptualization. **Edith M.G. van Esch:** .

## Funding

This work was supported by the Dutch Cancer Society [IKNL2019-12398].

## Declaration of competing interest

The authors declare that they have no known competing financial interests or personal relationships that could have appeared to influence the work reported in this paper.
