# Supplementary material for: Prognostic and therapeutic implications of clinical-radiologic discrepancy in parametrial invasion prior to primary radical hysterectomy in cervical cancer
Source: Gynecol Oncol Rep. 2025 Dec 9;63:102002. doi: 10.1016/j.gore.2025.102002 (PMC12769419; doi:10.1016/j.gore.2025.102002)
Supplement: Supplementary Data 1 [file mmc1.docx]

**SUPPLEMENTARY MATERIAL**

| **Supplementary Table S1. Multivariable analysis** | | | | | | |
| --- | --- | --- | --- | --- | --- | --- |
|  | **Overall survival** | | | **Disease-free survival** | | |
| Variables |  | | |  | | |
|  | HR | 95% CI | p-value | HR | 95% CI | p-value |
| Group |  |  |  |  |  |  |
| Consensus | 1.00 | Reference | | 1.00 | Reference | |
| Discrepancy | 0.94 | 0.55-1.62 | 0.82 | 1.00 | 0.60-1.66 | 0.99 |
| Age† | 1.04 | 1.03-1.06 | <0.001* | 1.02 | 1.00-1.03 | 0.012* |
| FIGO 2009 stage |  |  |  |  |  |  |
| I | 1.00 | Reference | | 1.00 | Reference | |
| II | 1.16 | 0.65-2.01 | 0.62 | 1.11 | 0.61-2.02 | 0.73 |
| Primary tumor size (mm)† | 1.03 | 1.02-1.05 | <0.001* | 1.03 | 1.01-1.04 | <0.001* |
| Pathological lymph node status |  |  |  |  |  |  |
| pN0 | 1.00 | Reference |  | 1.00 | Reference |  |
| pN1 | 1.32 | 0.81-2.15 | 0.26 | 0.84 | 0.54-1.31 | 0.45 |
| Histological subtype |  |  |  |  |  |  |
| Squamous cell carcinoma | 1.00 | Reference | | 1.00 | Reference | |
| Non-squamous cell carcinoma | 1.47 | 0.98-2.19 | 0.06 | 1.43 | 0.98-2.07 | 0.06 |
| LVSI |  |  |  |  |  |  |
| Absence | 1.00 | Reference |  | 1.00 | Reference |  |
| Presence | 1.80 | 1.17-2.77 | 0.008* | 3.00 | 1.97-4.58 | <0.001* |
| Pathological parametrial involvement |  |  |  |  |  |  |
| Absence | 1.00 | Reference |  | 1.00 | Reference |  |
| Presence | 1.54 | 0.84-2.82 | 0.17 | 1.34 | 0.71-2.55 | 0.37 |
| Positive surgical margins |  |  |  |  |  |  |
| Absence | 1.00 | Reference |  | 1.00 | Reference |  |
| Presence | 2.59 | 1.42-4.73 | 0.002* | 1.98 | 1.08-3.64 | 0.028* |
| *Statistically significant.  †Clinical tumor size, supplemented with pathological tumor size when missing  *Abbreviations:* FIGO, International Federation of Gynecology and Obstetrics; LVSI, lymphovascular space invasion | | | | | | |

| **Supplementary Table S2. Multivariable logistic regression analysis for the risk of adjuvant therapy** | | | |
| --- | --- | --- | --- |
| Variables |  | | |
|  | OR | 95% CI | p-value |
| Group |  |  |  |
| Consensus | 1.00 | Reference | |
| Discrepancy | 3.10 | 1.56-6.16 | 0.001* |
| Age | 1.01 | 0.99-1.03 | 0.32 |
| Primary tumor size (mm)† | 1.04 | 1.02-1.07 | <0.001* |
| Lymph node status |  |  |  |
| pN0 | 1.00 | Reference |  |
| pN1 | 161.30 | 62.80-414.30 | <0.001* |
| Depth of invasion |  |  |  |
| ≤5 mm | 1.00 | Reference | |
| >5 mm | 1.79 | 0.97-3.32 | 0.06 |
| LVSI |  |  |  |
| Absence | 1.00 | Reference |  |
| Presence | 3.32 | 1.95-5.66 | <0.001 |
| Positive surgical margins, yes |  |  |  |
| Absence | 1.00 | Reference |  |
| Presence | 53.37 | 10.61-268.36 | <0.001* |
| Pathological parametrial involvement |  |  |  |
| Absence | 1.00 | Reference |  |
| Presence | 11.56 | 3.06-43.75 | <0.001* |
| Constant | 0.01 | 0.00-0.03 | <0.001* |
| *Statistically significant.  †Clinical tumor size, supplemented with pathological tumor size when missing  *Abbreviations:* LVSI, lymphovascular space invasion | | | |
